# Is There a Link between Balance and Body Mass Composition in Children and Adolescents?

**DOI:** 10.3390/ijerph181910449

**Published:** 2021-10-04

**Authors:** Wojciech Rusek, Marzena Adamczyk, Joanna Baran, Justyna Leszczak, Grzegorz Inglot, Rafał Baran, Teresa Pop

**Affiliations:** 1 Rehabilitation Centre Rehamed-Center Sp. z o.o., 36-002 Tajęcina, Poland; rusekaw@interia.pl; 2 RehaKlinika Sp. z o.o., 36-021 Rzeszów, Poland; 3 Institute of Health Sciences, Medical College, University of Rzeszów, 35-315 Rzeszów, Poland; joannabaran.ur@gmail.com (J.B.); leszczakjustyna.ur@gmail.com (J.L.); popter@interia.pl (T.P.); 4 Natural and Medical Center for Innovative Research, 35-310 Rzeszów, Poland; 5 Institute of Medical Sciences, Medical College, University of Rzeszów, 35-315 Rzeszów, Poland; iglooo@interia.pl; 6 Individual Medical Practice Grzegorz Inglot, 36-060 Glogow Malopolski, Poland; 7 Solution-Statistical Analysis, 35-120 Rzeszów, Poland; rafal.barann@gmail.com

**Keywords:** balance, body composition, children, Zebris

## Abstract

School-age children are particularly prone to disturbances in body composition, because this is a period of intensive growth and a period in which correct habits are shaped, especially in relation to diet. This is why it is so important to diagnose emerging disorders early so as to implement therapeutic or educational activities. The aim of this study was to assess the relationship between the factors of body mass composition and body mass index (BMI), as well as the balance parameters in children and adolescents. The study group consisted of 1137 children aged 7 to 15. There were 559 girls and 578 boys among the subjects. The study used the Tanita 780 body mass composition analyser and the Zebris stabilometric platform. It was found that girls were characterized by a significantly higher content of adipose tissue (24.37% vs. 20.45%), while boys were characterized by a higher content of lean tissue (32.99% vs. 30.43%), muscle tissue (31.23% vs. 28.86%) and water (24.15% vs. 22.28%). Interestingly, the girls had better balance than their peers in all analyzed parameters (COF TTL.—616.72 vs. 661.50; CEArea—73.63 vs. 112.24; COF HD—3.44 vs. 4.23; COF VD—4.52 vs. 5.12). It turned out that among children in adolescence, a higher adipose tissue content and a higher BMI correlated with a smaller surface area (*p* < 0.05) defined by the center of gravity and smaller deviations of the center of gravity in the horizontal plane (*p* < 0.05). Sex and adolescence play an important role in differentiating both body composition and body balance. The results of this study allow us to conclude that children with higher BMI values have better balance. Due to the fact that these conclusions are inconsistent with those of other researchers, it will be worth continuing the research (e.g., on a different population group) in order to confirm the results and to draw far-reaching conclusions.

## 1. Introduction

Overweight and obesity has become a disease of civilization not only in Europe but also around the world [1]. Researchers report that those who are overweight and obese in childhood tend to maintain weight gain into adulthood. Obesity is a chronic condition that significantly shortens life as it is one of the factors in the occurrence of cardiovascular diseases [2]. The prevalence of obesity in childhood increases the risk of myocardial infarction, stroke, kidney diseases, sleep apnea, locomotor system diseases, neoplastic diseases and depression in adults [3,4]. It is concerning that the prevalence of overweight and obesity has continued to increase over the past 40 years. In some countries there has been an almost two-fold increase in obesity: in Israel it has increased from 5.8% to 11.9%, in Andorra from 6.2% to 12.8% and in Malta from 7.4% to 13.4%, while in 2016, during the economic crash in Europe, the prevalence of overweight increased by over 30% and obesity increased by over 10% [5].

The period of childhood is one in which intense changes take place in the human body. These changes affect not only external appearance, but also all of the systems and organs. In adolescence, weight gain is on average 14 kg in girls and 15 kg in boys [6]. The epidemic of overweight and obesity is becoming more and more noticeable and requires decisive action that would lead to weight reduction by increasing physical activity and shaping proper eating habits. According to the World Health Organization (WHO), being underweight or overweight are factors that threaten human health [7]. Therefore, it is so important to prevent overweight and obesity, which should start as early as childhood [8,9,10,11,12]. In the literature, authors have described the prevention programs of the members of the European Childhood Obesity Group (ECOG) who have undertaken the dissemination of projects promoting a healthy lifestyle and weight control with the participation of the media and members of the medical profession [13,14,15,16].

Another very important aspect in the development of a child is the mastery of Fundamental Movement Skills (FMS), which are considered the basis of an active lifestyle [17]. One of the aspects that is inextricably linked with motor development and FMS is balance, which is most often defined as the ability to maintain an upright posture and to keep the centre of gravity within the limits of the base of support. The development of balance in the first year of life leads to the achievement of an independent bipedal posture. Then, until about 7–8 years of age, the range of swaying rises along with a constantly increasing speed and frequency. After this critical period, the stability parameters gradually improve until a stable body posture is obtained at the age of about 15 years [18,19,20]. In children with obesity, there is a reduction in Fundamental Movement Skills and motor coordination, which means that these children have reduced physical activity already in early childhood [21,22].

Another unfavorable factor is the contemporary lifestyle and civilization challenges that have had a negative impact on the physical activity of children and adolescents, increasing the tendency towards sedentary lifestyles [23,24]. The guidelines of the American Academy of Pediatrics Policy Statement state that a sedentary lifestyle exceeding 1.5 h a day poses a risk of obesity in children aged 4 to 9 [25]. Long periods of time spent in front of the TV or computer, the use of motorized transport, poor nutrition and a lack of regular physical activity contribute to the poor physical condition of children [26,27]. It is stated that children aged 5 to 17 should spend 60 min a day on moderate or vigorous physical activity (MVPA) such as physical education lessons, housework, sports and games and recreational activities [28]. Growing up is a time for shaping health behaviours in children, most of which remain unchanged throughout their lives. The younger generation does not have sufficient knowledge about proper nutrition, or about other health-promoting behaviours. This knowledge should be obtained first and foremost from parents, but also through school, and reliable information should be provided by the media and the press. Therefore, the issue of metabolic disorders and abnormal body posture should be a key focus point not only in medicine or science, but also in the education sector. It should provide children and their parents with information about correct health-promoting behaviour patterns and the consequences of not following them [29,30].

There is a large amount of information in the literature on health disorders caused by obesity, but little information about the relationship between balance and body weight composition. To fill this scientific gap, this study was conducted to assess body weight composition and its impact on balance by sex and age.

## 2. Materials and Methods

### 2.1. Participants

Before starting the study, the sample size was calculated on the basis of the total number of children living in the Trzebownisko municipality (*n* = 2302), with a 95% confidence level and a confidence interval of 0.05. It was calculated that the minimum sample size should be 329. The study group consisted of 1137 children aged 7 to 15 from primary or lower secondary school in the municipality, which constitutes 50% of the population of the community. The research was conducted in the period from September 2016 to February 2020. Five primary and lower secondary schools were randomly selected from among the nine schools located in the given rural region in the south-eastern part of the country where the study was conducted. The research was conducted in the nursing offices of the relevant educational institutions. To ensure the reliability of the measurements, all subjects, in a fasted state, were examined in the morning by the same qualified members of the team.

The inclusion criteria were children aged from 7 to 15 and the consent of the parent/legal guardian and the child to participate in the study.

The exclusion criteria were children under 7 or over 15 years of age, lack of consent of the parent/legal guardian or child to participate in the study, diseases or injuries affecting body posture and balance, a difference in lower limb length greater than 5 mm, menstruation (on the day of testing), epilepsy, an implanted pacemaker and metal implants.

**Figure 1:**
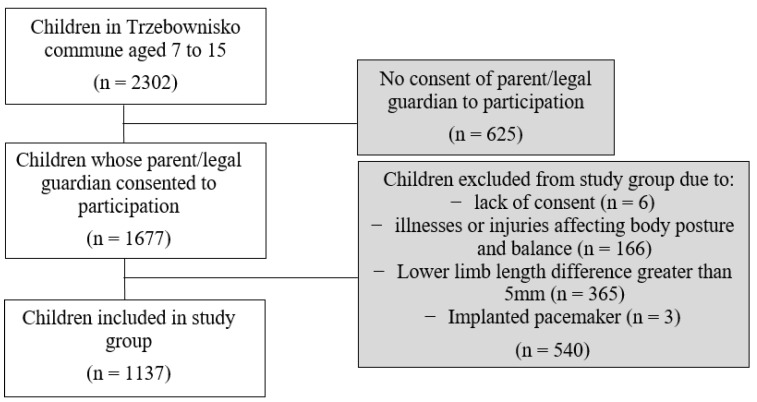
Flow chart of participants.

Inclusion in and exclusion from the study group was carried out according to the flowchart (Figure 1).

### 2.2. Procedures

The examination of each child consisted of:

1. Measurement of body height with the PORTSTAND 210 portable stadiometer. During the test, each participant stood upright, wearing underwear and no shoes. Body height was measured under standard conditions with an accuracy of 0.1 cm and was used to calculate the BMI.
2. Assessment of body mass composition using the Tanita 780 analyser. This is a device which serves to estimate the components of body mass, based on the principles of bioelectrical impedance. Subjects had not eaten for at least 2 h before the measurement and had been asked to empty their bladders before the measurements began. Subjects stood bare-footed on metal sole plates attached to electrodes and were also asked to hold a handle-shaped electrode in each hand. The current flowing between the electrodes encounters more resistance when passing through adipose tissue than lean body mass and water, which allows for an accurate analysis of the composition of the body mass [31,32]. As a result of the body mass composition analysis, the following parameters were assessed: percentage body fat content (FatP) [%], body fat content (FatM) [kg], lean tissue content (FFM) [kg], muscle mass (PMM) [kg] and total body water (TBW) [kg].
3. BMI calculations based on body weight and height (underweight, normal weight, overweight and obesity) were classified according to sex and age percentile grids [33].
4. Body balance assessments on the Zebris stabilometric platform. The test consisted of maintaining a standing position on the platform for 20 s. The examined person stood freely, without shoes, with the upper limbs lowered and his eyes focused on a black dot on the wall at eye level. As a result of the study, the following parameters were obtained: distance covered by the projection of the center of force of the feet onto the platform (COF TTL) [mm], lateral inclination of the projection of the center of force of the feet onto the platform (COF HD) [mm], anterior–posterior projection of the center of force of the feet onto the platform (COF VD) [mm], area of the ellipse as shown by the projection of the center of force of the feet onto the platform (CEArea) [mm^2^] and the symmetry index calculated on the basis of the percentage load on the lower limbs, dividing the greater value by the lower one. The correct range for the symmetry index is between 1.00 and 1.15 [34].

The research was carried out by a research team consisting of 4 people. Each team member was assigned to one piece of research equipment. Therefore, the first researcher took all of the height measurements with the PORTSTAND 210 stadiometer in each study. The second researcher was responsible for assessing the body weight composition with the Tanita 780 analyser. The third researcher assessed body balance on the Zebris stabilometric platform. The fourth researcher conducted an interview, which consisted of the subjects giving their date of birth, place of residence, sex and contraindications for the examination, and also familiarized the subjects with what the examination would involve. One researcher was needed to operate each piece of research equipment; therefore, to ensure the precision and reliability of the research and the minimal risk of error during the test, the same researcher always performed tests on the same piece of research equipment.

Bearing in mind that the studied group included some children in adolescence, this issue was taken into account in the analysis. Puberty is a time when numerous sex-specific changes occur in a young body. In girls these include a greater increase in adipose tissue, especially in the thighs and buttocks, and in boys an increase in muscle mass [35,36].

In this study, Tanner’s puberty cut-offs were considered for girls ≥ 10 years of age and for boys ≥ 12 years of age [37]. This division has been cited by numerous researchers [38,39,40,41].

Participants were informed about the course of the study, and written consent was obtained from school principals and parents or legal guardians of the studied children. The children and their parents were informed that the tests results would be anonymized and added to a larger data pool. The research was approved by the Bioethics Committee of the University of Rzeszów, no. 2016/06/28 of 28 June 2016.

### 2.3. Statistical Analysis

Possible correlations between the variables were analyzed in two stages. In the first step, the assumption about the normality of the distribution of variables was verified using the Kolmogorov–Smirnov test. It was confirmed that the distributions of almost all variables were not normal. Therefore, it was decided to consider the correlation between the variables using the nonparametric Spearman’s Rho correlation coefficient. Two non-parametric tests were also used to characterize the group: Mann–Whitney (for two independent variables) and Kruskal–Wallis (for variables with more than two independent categories). The significance level was assumed at *p* < 0.05. All of the calculations and statistical analyses were computed using STATISTICA ver. 10.0 (StatSoft).

## 3. Results

The entire study population was presented as the study group. It included 1137 children aged 7 to 15. Girls constituted 49.2% of the subjects (*n* = 559), and boys 50.8% (*n* = 578). The characteristics were presented by means of descriptive statistics such as mean value, standard deviation, lower and upper quartile, median, minimum and maximum. The parameters of body mass composition (Body fat content—FatP in%, Body fat content—FatM in kg, Lean tissue mass—FFM in kg, Water content—TBW in kg and Muscle tissue mass—PMM in kg) and the balance of the subjects were analyzed. (CEArea [mm^2^]—area of the ellipse as shown by the projection of the center of force of the feet onto the platform; COF TTL [mm]—distance covered by the projection of the center of force of the feet onto the platform; COF HD [mm]—lateral inclination of the projection of the center of force of the feet onto the platform; COF VD [mm]—anterior–posterior projection of the center of force of the feet onto the platform.) The detailed characteristics of the subjects are presented in Table 1.

Significant differences were found in the values of body weight and balance parameters between girls and boys, as shown in Table 2. Girls were characterized by a significantly higher content of adipose tissue (expressed in % and kg), while boys were characterized by a higher content of lean tissue, muscle tissue and water. Interestingly, the girls had better balance than their peers in all analyzed parameters.

Next, the correlation between body mass composition and balance parameters was analyzed, taking into account the age of the subjects. It turned out that among children in adolescence, a higher adipose tissue content and a higher BMI correlated with a smaller surface area defined by the center of gravity and smaller deviations of the center of gravity in the horizontal plane. In the case of the path length determined by the center of gravity, higher values of BMI and body mass components correlated with a shorter path length in both pre-pubertal and adolescent children. It was also noticed that lower values of the symmetry coefficient were associated with a higher content of all body components in children in adolescence. Detailed data of Spearman’s rho with regard to the level of significance are presented in Table 3.

Since puberty differs between girls and boys, the above analysis was performed taking into account the sex of the subjects. In the case of the path length, negative correlations were also obtained with the body components, regardless of the sex and age of the subjects. On the other hand, differentiation was noticed in the case of the ellipse surface area, where in girls there was a negative relationship with the content of adipose tissue in adolescence, and in boys with lean tissue, muscle and water before puberty. On the other hand, the size of the horizontal deflections was determined by the age before puberty in boys, and by the puberty period in girls (Table 4).

## 4. Discussion

The aim of the study was to evaluate body mass composition and its influence on balance. Additionally, balance was assessed taking into account the sex and age of the subjects. Conducting research on children and adolescents, showing possible disorders or pathologies and looking for and finding solutions to the observed problems is crucial for maintaining the health of a given population. The present study was conducted on a fairly large group of children who had not previously been diagnosed with any musculoskeletal disorders. In addition, the study did not cover people with neurological diseases, skeletal deformities, after orthopedic surgery or with other disorders that may affect body posture or balance. Nevertheless, some of the subjects showed features of incorrect body posture and balance disorders.

The authors’ own research showed that girls were characterized by a significantly higher content of adipose tissue (expressed in % and kg), while boys were characterized by a higher content of lean tissue, muscle tissue and water. Interestingly, the girls had better balance than their peers in all analyzed parameters. Leskinen et al. in their studies also observed a difference in the occurrence of body mass components due to sex. They showed that as early as 3 years of age one can observe a difference between the amount of lean tissue between boys and girls (boys 11.7 kg, girls 11.3 kg; *p* < 0.001) [42]. Sen and Mondal also confirmed the difference in the occurrence of body mass components in terms of sex. Statistically significant differences between the sexes were observed in FM and FFM (*p* < 0.05) [43].

The available literature includes scientific articles on the relationship between body mass composition and BMI and balance parameters. McGraw et al., after examining 20 boys (10 obese and 10 with normal body weight) aged 8–10 years, found that the obese boys showed significantly greater areas of medial/lateral sway and swing than the non-obese boys [44]. Maślanko et al. also investigated this relationship by carrying out a balance assessment based on the Biodex system among 166 children aged 7 to 18. The aim of their analysis was to compare the stability of children of normal body weight with obese children. Studies have shown that in all balance tests on an unstable platform, obese children perform significantly worse compared to their non-obese peers. However, the results showed no differences when a static platform was used to compare the stability of obese children with children of normal body weight [45]. Lungren et al. conducted their research among 246 boys and 190 girls, aged 6–12 years, assessing neuromuscular fitness such as muscle strength, vertical jump height (VJH) and standing unilateral balance, in pre-pubertal children. Their research found that anthropometry, muscle strength and VJH in both sexes showed an improved performance with advancing age (all correlations *p* < 0.01) but there were no consistent sex differences across the age groups (all non-significant). In boys and girls, the ratio of muscle strength/muscle mass showed significantly higher values with higher ages (*p* < 0.01 for both sexes), but with no consistent sex difference. The postural control tests also showed significantly better performance with higher ages in both boys and girls (both *p* < 0.01) [46]. A similar relationship was described in research by Guan et al., who stated that overweight, obesity, and body composition of female pupils in Chongqing city, China, correlated with early pubertal timing, while overweight and lean body mass of male pupils correlated with early pubertal timing [47]. Our own research confirms the difference in the examined parameters before and during adolescence. It has been shown that among children in adolescence, a higher adipose tissue content and a higher BMI correlated with a smaller surface area defined by the center of gravity and smaller deviations of the center of gravity in the horizontal plane. In the case of the path length determined by the center of gravity, higher values of BMI and body mass components correlated with a shorter path length in both pre-pubertal and adolescent children. It was also noticed that lower values of the symmetry coefficient were associated with a higher content of all body components in children in adolescence.

Condon and Cremin conducted six clinical balancing tests among 534 children aged 4 to 15 years. On the basis of the conducted research, they found that correlations regarding body weight and balance were present only in selected tests, in the younger age groups (6–7 years) [48]. In their research, Guzmán-Muñoz et al. assessed the relationship between the anthropometric profile and the postural and dynamic balance in children aged 6 to 9 years. They surveyed 158 pupils (88 boys and 70 girls). The variables of the anthropometric profile studied were body mass, bipedal stature, body mass index (BMI), waist circumference (WC), waist-to-hip ratio (WHR), sum skin-folds, body composition and somatotype. In addition, the static and dynamic postural balance were measured through posturography and the Y-Balance Test, respectively. The authors found that children with greater obesity and/or predominance of the endomorphic component performed worse on static and dynamic posture balance tests, respectively. Anthropometric measurements were correlated with the results of postural balance tests. They showed moderate positive correlations between static postural balance, mainly with eyes closed, and BMI, PC, sum of skin folds, fat mass, and endomorphism. With regard to dynamic postural balance, moderate negative correlations were observed between the performance of the Y-balance test and body weight, bipedal posture, BMI, sum of skin folds, fat mass, skin mass and endomorphism [49]. This is consistent with the results of the authors’ own research.

In turn, the studies of Pagnotti et al. showed a different trend. In their studies, they showed that obese subjects showed a greater displacement of COP at higher AP speeds compared to non-obese subjects, suggesting that subjects with clinical obesity exhibit greater instability than non-obese subjects [50].

In the present study, the analysis of the child population confirmed the significant influence of the BMI index on body stability. It has been shown that higher BMI values among children translate into better body balance. This was visible in the form of the decreasing area of the ellipse as shown by the projection of the center of force of the feet onto the platform, the shorter distance covered by the projection of the center of force of the feet onto the platform, the decreasing deflection of the lateral projection of the center of force of the feet onto the platform and the decreasing index of symmetry. Similar relationships concerned the components of body weight. It was found that children with a higher percentage of adipose tissue mass, a higher content of adipose tissue, lean tissue and muscle tissue and a higher water content in the body had a smaller ellipse surface area as defined by the projection of the center of force of the feet onto the platform, the shorter distance covered by the projection of the center of force of the feet onto the platform, the lower inclination of the lateral projection of the center of force of the feet onto the platform and lower values of the symmetry index. The most intriguing result of this study, then, was the finding of a strong relationship between high BMI and body balance. It turned out that overweight and obese children have better stability parameters than children with normal body weight, which is confirmed by many studies [51,52] but there are also studies that do not agree with our results [53,54,55,56,57,58].

When qualifying children for the study, it was assumed that the study would be conducted among children who had not previously been diagnosed with any musculoskeletal disorders. In addition, the study did not cover people with neurological diseases, skeletal deformities, after orthopedic surgery or with other disorders that may affect body posture or balance. Condon and Cremin, in their research on the norms of static balance in children, stated that the assessment of balance should take into account ankle and foot injuries [48]. Similarly, in their research, Gibble et al. described the relationship between lower limb injuries and the assessment of balance. They reported that there are many methods, tests and devices to assess static and dynamic balance, and in their research they stated that the Star Excursion Balance Test (SEBT) has been shown to be a reliable measure and has validity as a dynamic test to predict risk of lower extremity injury, to identify dynamic balance deficits in patients with a variety of lower extremity conditions and to be responsive to training programs in both healthy people and people with injuries to the lower extremity [59]. Samaan et al. assessed the effects of ACL reconstruction on lower extremity joint mechanics during the performance of the Star Excursion Balance Test (SEBT) and the Single Leg Hop (SLH) are limited. In their research they noticed that despite normal functional efficiency during SEBT and SLH, the athlete showed altered mechanics of the joints of the lower limbs during both of these tasks [60].

Cote et al. reported that the stability of the posture is also influenced by the type of foot, both in static and dynamic conditions [61]. Tsai et al. also found a relationship between foot type and balance, stating that individuals with pronated feet or supinated feet have poorer postural control than individuals with neutral feet [62]. Yi et al. assessed the differences between static and dynamic balance among three foot types and the changes in postural balance while wearing typical athletic shoes. The showed that the medial longitudinal arch of the foot affects postural balance. Typical athletic shoes improve postural balance regardless of foot type. However, the pronated and supinated foot groups still had a lower dynamic postural balance compared with the neutral foot group, even when wearing athletic shoes. People with pronated and supinated feet may need additional interventions, such as foot orthoses or balance training [63].

As Eid et al. wrote, children with Down’s syndrome (DS) often have greater postural sway and delays in motor development [64]. Geuze assessed postural control in children with developmental coordination disorder and wrote that the group of children with DCD and balance problems, however, showed a weaker coupling between EMG and corrective force compared with control children, indicating non-optimal balance control [65]. Dewar et al., examining body posture and balance among children with cerebral palsy (CP), also found the need for increased exercise to improve body balance [66]. In their research, Mickle et al. reported that age and gender also influence the control of the equilibrium. They noticed that boys displayed greater sway than the girls for all conditions, although only the single limb stance scores were significantly different between the two groups (boys: 632 ± 323 mm; girls: 456 ± 338 mm; *p* = 0.04). Eight-year-old children displayed significantly greater sway than the older children during the two dual limb stance conditions, whereas the 8-year-old children performed significantly poorer during the single limb condition than the 10-year-old children [67]. Plandowska et al. investigated the postural stability of 5-year-old girls and boys of different height and noticed that sex-related differences were found during a natural stance for all COP parameters. Girls maintained a two-legged standing position with a lower sway velocity and a smaller range of sway than their male counterparts. Normal- and tall-statured girls demonstrated better postural stability significantly more often than boys [68].

Due to the influence of neurological disorders, the musculoskeletal system, malformations of children, surgery, orthopedic procedures and other disorders that affect the balance of the body, children without the above-mentioned disorders were included in the study group.

The increase in the prevalence of overweight and obesity among children and adolescents drew the researchers’ attention to additional health complications that may occur in this population. The research methodology used in this work allowed for a detailed assessment of the impact of body mass composition and BMI on the balance of children and adolescents.

### 4.1. Limitations of This Study

The scope of this study has been narrowed down to one area which may be a limitation of the study. Future research could be planned in the form of a controlled experiment, extended to other regions of the country. Such a procedure will allow for a more detailed understanding of the cause-and-effect relationships regarding the impact of body mass composition and body mass index (BMI) on the balance parameters in children and adolescents. In subsequent studies, a longitudinal study may be planned, comparing the body composition–balance relationship in children before puberty and then in adolescence.

### 4.2. Clinical Implication

The obtained results are important for the practice of doctors, physiotherapists or dieticians. They make it possible to state that boys, despite their natural tendency to move more than girls, have worse balance than girls.

In addition, it has been confirmed that girls have adipose tissue in a greater percentage, especially in adolescence, which in turn gives a signal to control before the occurrence of excess body weight.

On the basis of the conducted research, it was found that in the analyzed group, people with higher BMI values had better equilibrium parameters. It is worth continuing the research to confirm this thesis among other populations.

## 5. Conclusions

The conducted research proves that there is a correlation between the composition of body mass and balance in humans. Sex and adolescence play an important role in differentiating both body composition and body balance. Girls in adolescence are characterized by a higher content of adipose tissue and better balance parameters than boys, who, on the other hand, are characterized by a greater content of muscle tissue, which also correlates with better stability. The results of this study allow us to conclude that children with higher BMI values have a better balance. Due to the fact that these conclusions are inconsistent with those of other researchers, it is worth continuing the research (e.g., on a different population group) in order to confirm the research and draw far-reaching conclusions.

## Figures and Tables

**Table 1:** Characteristics of the study group.

Variables	Mean	SD	Q_1_	Me	Q_3_	Min	Max
Age (years)	10.53	2.49	8.00	10.00	13.00	7	15
Height (cm)	147.15	15.76	134.50	146.50	160.00	109.00	189
Weight (kg)	41.39	14.61	29.60	38.80	50.85	15.70	106.50
BMI (kg/m^2^)	18.54	3.62	15.80	17.85	20.70	11.10	34.10
FatP (%)	22.38	6.13	17.80	21.50	26.30	6.70	47.40
FatM (kg)	9.64	5.42	5.60	8.30	12.30	1.50	45.70
FFM (kg)	31.73	10.33	23.35	29.90	38.25	13.80	65.70
PMM (kg)	30.06	9.84	22.10	28.30	36.25	13.00	62.40
TBW (kg)	23.23	7.56	17.10	21.90	28.00	10.10	48.10
COF TTL (mm)	639.48	214.09	476.55	611.90	766.75	196.70	1510.50
COF HD (mm)	3.84	3.17	2.30	3.10	4.50	0.80	64.50
COF VD (mm)	4.83	2.48	3.20	4.30	5.80	0.90	21.60
CEArea (mm^2^)	93.26	160.37	34.25	57.60	102.95	4.40	3864.60
Symmetry index	1.25	0.43	1.07	1.16	1.30	1.00	11.50

BMI—Body mass index; CEArea [mm^2^]—area of the ellipse as shown by the projection of the center of force of the feet onto the platform; COF TTL [mm]—distance covered by the projection of the center of force of the feet onto the platform; COF HD [mm]—lateral inclination of the projection of the center of force of the feet onto the platform; COF VD [mm]—anterior–posterior projection of the center of force of the feet onto the platform. FatP—body fat content in %, FatM—body fat content in kg, FFM—lean tissue mass in kg, Max—maximum value; Me—median; Mean—average value; Min—minimum value; PMM—muscle tissue mass in kg, Q_1_—lower quartile; Q_3_—higher quartile; SD—standard deviation; TBW—total body water in kg.

**Table 2:** Comparison of results of body weight composition, BMI and balance measurements by gender.

Variable	Females		Males		*p*
	Mean	SD	Mean	SD	
BMI	18.49	3.67	18.57	3.58	0.775
FatP (%)	24.37	5.52	20.45	6.07	<0.001
FatM (kg)	10.44	5.57	8.87	5.16	<0.001
FFM (kg)	30.43	8.83	32.99	11.47	0.007
TBW (kg)	22.28	6.46	24.15	8.39	0.007
PMM (kg)	28.86	8.39	31.23	10.94	0.010
CEArea (mm^2^)	73.63	74.85	112.24	210.91	<0.001
COF TTL (mm)	616.72	203.33	661.50	221.96	0.002
COF HD (mm)	3.44	2.05	4.23	3.93	<0.001
COF VD (mm)	4.52	2.17	5.12	2.71	0.001
Symmetry index	1.23	0.52	1.27	0.32	<0.001

BMI—Body mass index; CEArea [mm^2^]—area of the ellipse as shown by the projection of the center of force of the feet onto the platform; COF TTL [mm]—distance covered by the projection of the center of force of the feet onto the platform; COF HD [mm]—lateral inclination of the projection of the center of force of the feet onto the platform; COF VD [mm]—anterior–posterior projection of the center of force of the feet onto the platform. FatP—body fat content in %, FatM—body fat content in kg, FFM—lean tissue mass in kg, Mean—average value; *p*—statistical significance; SD—standard deviation; PMM—muscle tissue mass in kg, TBW—total body water in kg.

**Table 3:** Correlation between the composition of body mass, BMI and balance parameters (Rho value).

Body Mass Composition Factor	n	CEArea [mm^2^]	COF TTL [mm]	COF HD [mm]	COF VD [mm]	Symmetry Index
**Body fat content (FatP) [%]**						
Before puberty	592	−0.026	−0.443 **	−0.044	0.003	−0.105 *
Puberty	545	−0.113 *	−0.351 **	−0.137 *	−0.056	−0.072
**Body fat content (FatM) [kg]**						
Before puberty	592	−0.039	−0.588 **	−0.063	0.004	−0.098 *
Puberty	545	−0.089 *	−0.395 **	−0.127 *	−0.032	−0.129 *
**Lean tissue mass (FFM) [kg]**						
Before puberty	592	−0.049	−0.570 **	−0.065	−0.005	−0.069
Puberty	545	0.006	−0.217 **	−0.038	0.028	−0.115 *
**Muscle tissue mass (PMM) [kg]**						
Before puberty	592	−0.051	−0.571 **	−0.066	−0.007	−0.070
Puberty	545	0.006	−0.217 **	−0.038	0.028	−0.115 *
**Water content (TBW) [kg]**						
Before puberty	592	−0.050	−0.569 **	−0.065	−0.005	−0.068
Puberty	545	0.006	−0.218 **	−0.038	0.028	−0.115 *
**BMI**						
Before puberty	592	−0.024	−0.533 **	−0.051	0.026	−0.077
Puberty	545	−0.088 *	−0.399 **	−0.116 *	−0.041	−0.120 *

** *p* < 0.001, * *p* < 0.05. BMI—Body mass index; CEArea [mm^2^]—area of the ellipse as shown by the projection of the center of pressure of the feet on the platform; COF TTL [mm]—distance covered by the projection of the center of pressure of the feet onto the platform; COF HD [mm]—inclination of the projection of the center of pressure of the feet onto the platform sideways; COF VD [mm]—forward–rearward projection of the center of pressure of the feet onto the platform.

**Table 4:** Correlation between body mass composition, BMI and gender balance parameters (Rho value).

Body Mass Composition Factor		n	CEArea [mm^2^]	COF TTL [mm]	COF HD [mm]	COF VD [mm]	Symmetry Index
**Body fat content (FatP) [%]**							
Girls	Before puberty	213	0.013	−0.419 **	−0.028	0.049	−0.112
	Puberty	346	−0.136 *	−0.425 **	−0.137 *	−0.102	−0.086
Boys	Before puberty	379	0.026	−0.464 **	0.010	0.038	−0.052
	Puberty	199	0.040	−0.246 **	−0.048	0.106	−0.077
**Body fat content (FatM) [kg]**							
Girls	Before puberty	213	−0.010	−0.526 **	−0.026	0.030	−0.107
	Puberty	346	−0.124 *	−0.464 **	−0.142 *	−0.085	−0.156 *
Boys	Before puberty	379	−0.050	−0.619 **	−0.078	−0.005	−0.090
	Puberty	199	−0.003	−0.269 **	−0.084	0.076	−0.103
**Lean tissue mass (FFM) [kg]**							
Girls	Before puberty	213	−0.030	−0.541 **	−0.004	−0.009	−0.103
	Puberty	346	−0.069	−0.378 **	−0.110 *	−0.033	−0.186 *
Boys	Before puberty	379	−0.139 *	−0.628 **	−0.168 *	−0.063	−0.063 *
	Puberty	199	−0.021	−0.123	−0.061	0.025	−0.064
**Muscle tissue mass (PMM) [kg]**							
Girls	Before puberty	213	−0.030	−0.541 **	−0.004	−0.009	−0.103
	Puberty	346	−0.069	−0.379 **	−0.111 *	−0.033	−0.186 *
Boys	Before puberty	379	−0.139 *	−0.627 **	−0.168 *	−0.063	−0.122 *
	Puberty	199	−0.021	−0.122	−0.061	0.024	−0.065
**Water content (TBW) [kg]**							
Girls	Before puberty	213	−0.030	−0.541 **	−0.004	−0.009	−0.102
	Puberty	346	−0.069	−0.379 **	−0.111 *	−0.034	−0.185 *
Boys	Before puberty	379	−0.139 *	−0.627 **	−0.168 *	−0.063	−0.123 *
	Puberty	199	−0.022	−0.123	−0.062	0.025	−0.064
**BMI**							
Girls	Before puberty	213	−0.020	−0.485 **	−0.046	0.029	−0.130
	Puberty	346	−0.132 *	−0.470 **	−0.145 *	−0.095	−0.142 *
Boys	Before puberty	379	−0.048	−0.568 **	−0.075	0.005	−0.066
	Puberty	199	−0.029	−0.304 **	−0.085	0.044	−0.089

** *p* < 0.001, * *p* < 0.05. BMI—Body mass index; CEArea [mm^2^]—area of the ellipse as shown by the projection of the center of pressure of the feet on the platform; COF TTL [mm]—distance covered by the projection of the center of pressure of the feet onto the platform; COF HD [mm]—inclination of the projection of the center of pressure of the feet onto the platform sideways; COF VD [mm]—forward–rearward projection of the center of pressure of the feet onto the platform.

## Data Availability

The data presented in this study are available on request from the corresponding author.
